# *N*-(3-Chloro­phen­yl)-*N*′-(2-methyl­phenyl)succinamide monohydrate

**DOI:** 10.1107/S1600536811014942

**Published:** 2011-04-29

**Authors:** B. S. Saraswathi, Sabine Foro, B. Thimme Gowda

**Affiliations:** a Department of Chemistry, Mangalore University, Mangalagangotri 574 199, Mangalore, India; b Institute of Materials Science, Darmstadt University of Technology, Petersenstrasse 23, D-64287 Darmstadt, Germany

## Abstract

In the title compound, C_17_H_17_ClN_2_O_2_·H_2_O, the dihedral angles formed by the aromatic rings of the chloro­benzene and methyl­benzene groups with the mean planes of the attached NH–C(O)–CH_2_ fragments are 9.4 (4) and 62.9 (2)°, respectively. In the crystal, mol­ecules are packed into layers parallel to the *bc* plane by O—H⋯O and N—H⋯O hydrogen-bond inter­actions.

## Related literature

For our study on the effects of substituents on the structures of *N*-(ar­yl)amides, see: Gowda *et al.* (2004 ▶); Saraswathi *et al.* (2011**a* ▶,b* ▶). For the oxidative strengths of *N*-chloro-*N*-aryl­sulfonamides, see: Gowda & Kumar (2003 ▶).
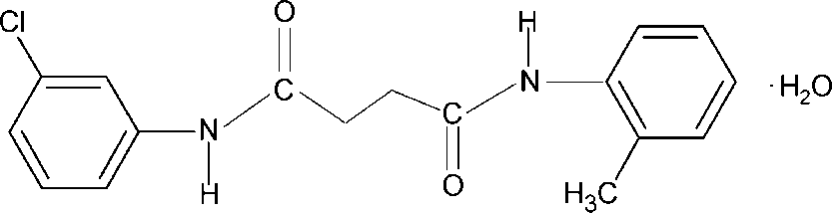


## Experimental

### 

#### Crystal data

C_17_H_17_ClN_2_O_2_·H_2_O
*M* *_r_* = 334.79
Monoclinic, 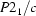

*a* = 14.875 (4) Å
*b* = 13.908 (3) Å
*c* = 8.088 (2) Å
β = 90.11 (2)°
*V* = 1673.3 (7) Å^3^
*Z* = 4
Mo *K*α radiation
μ = 0.24 mm^−1^
*T* = 293 K
0.44 × 0.12 × 0.08 mm

#### Data collection

Oxford Diffraction Xcalibur diffractometer with a Sapphire CCD detector
Absorption correction: multi-scan (*CrysAlis RED*; Oxford Diffraction, 2009 ▶) *T*_min_ = 0.900, *T*_max_ = 0.981
6273 measured reflections
3080 independent reflections
1436 reflections with *I* > 2σ(*I*)
*R*_int_ = 0.074

#### Refinement

*R*[*F* ^2^ > 2σ(*F* ^2^)] = 0.115
*wR*(*F* ^2^) = 0.285
*S* = 1.14
3080 reflections
221 parameters
5 restraints
H atoms treated by a mixture of independent and constrained refinement
Δρ_max_ = 0.66 e Å^−3^
Δρ_min_ = −0.27 e Å^−3^

### 

Data collection: *CrysAlis CCD* (Oxford Diffraction, 2009 ▶); cell refinement: *CrysAlis RED* (Oxford Diffraction, 2009 ▶); data reduction: *CrysAlis RED*; program(s) used to solve structure: *SHELXS97* (Sheldrick, 2008 ▶); program(s) used to refine structure: *SHELXL97* (Sheldrick, 2008 ▶); molecular graphics: *PLATON* (Spek, 2009 ▶); software used to prepare material for publication: *SHELXL97*.

## Supplementary Material

Crystal structure: contains datablocks I, global. DOI: 10.1107/S1600536811014942/rz2582sup1.cif

Structure factors: contains datablocks I. DOI: 10.1107/S1600536811014942/rz2582Isup2.hkl

Supplementary material file. DOI: 10.1107/S1600536811014942/rz2582Isup3.cml

Additional supplementary materials:  crystallographic information; 3D view; checkCIF report

## Figures and Tables

**Table 1:** Hydrogen-bond geometry (Å, °)

*D*—H⋯*A*	*D*—H	H⋯*A*	*D*⋯*A*	*D*—H⋯*A*
N1—H1*N*⋯O3^i^	0.86 (2)	2.08 (2)	2.940 (7)	175 (6)
N2—H2*N*⋯O2^ii^	0.86 (2)	2.25 (4)	2.991 (7)	145 (6)
O3—H31*O*⋯O2	0.85 (2)	2.04 (3)	2.861 (6)	163 (8)
O3—H32*O*⋯O1^iii^	0.84 (2)	2.05 (3)	2.877 (6)	171 (9)

Symmetry codes: (i) 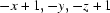; (ii) 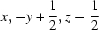; (iii) 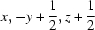.

**Fig. 1.**
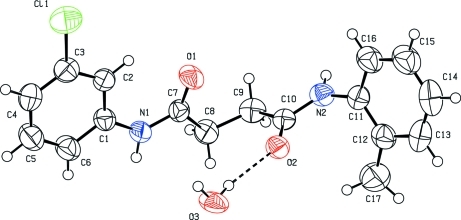
The asymmetric unit of the title compound with displacement ellipsoids drawn at the 50% probability level. The intermolecular O—H···O hydrogen bond involving the water molecule is drawn as a dashed line.

**Fig. 2.**
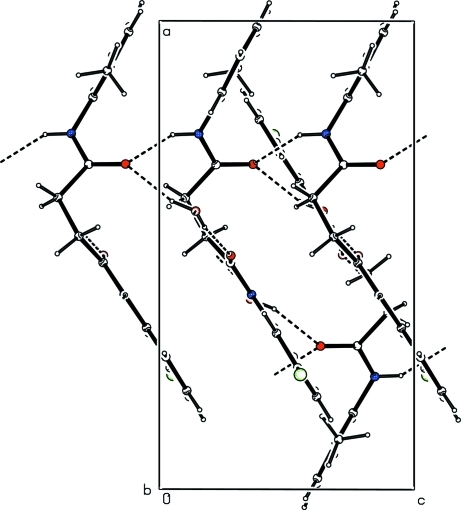
A partial packing diagram of the title compound viewed along the b axis, showing the hydrogen-bonding scheme with dashed lines.

## supplementary crystallographic information

### Comment

The amide and sulfonamide moieties are important constituents of many biologically important compounds. As a part of studying the substituent effects on the structures and other aspects of this class of compounds (Gowda & Kumar, 2003; Gowda *et al.*, 2004; Saraswathi *et al.*, 2011**a*,b*), in the present work, the structure of *N*-(3-chlorophenyl)-*N*-(2-methylphenyl)-succinamide monohydrate is reported (Fig. 1). The conformations of the N—H and C═O bonds in the C—NH—C(O)—C—C—C(O)—NH—C fragment are *anti* to each other and the amide O atom is *anti* to the H atoms attached to the adjacent C atoms. Further, the conformations of the N—H bonds in the amide fragments are *anti* to the *meta*-chloro or the *ortho*-methyl groups in the respective adjacent benzene rings, similar to the *anti* conformations observed with respect to the *ortho*-methyl groups in *N*,*N*'-bis(2-methylphenyl)succinamide (II; Saraswathi *et al.*, 2011*a*) and the *meta*-chloro groups in *N*,*N*'-bis(3-chlorophenyl)-succinamide (III; Saraswathi *et al.*, 2011*b*).

The dihedral angle between the 3-chlorobenzene ring and the adjacent NH—C(O)—CH_2_ group is 9.4 (4)° and that between the 2-methylbenzene ring and the adjacent NH—C(O)—CH_2_ group is 62.9 (2)°, compared to the values of 62.1 (2)° for that between the benzene ring and the NH—C(O)—CH_2_ group in the two halves of (II), and 32.8 (1)° in (III)

The torsion angles C1—N1—C7—C8 and C11—N2—C10—C9 are -173.0 (6)° and -177.9 (6)°, in contrast to the value of 69.5 (7)° for the torsion angle C7—C8—C9—C10.

In the crystal packing, molecules are linked by N1—H1N···O3, N2—H2N···O2, O3—H31O···O2 and O3—H32O···O1 hydrogen bonds (Table 1; Fig. 2) to form layers parallel to the *bc* plane.

### Experimental

Succinic anhydride (0.01 mol) in toluene (25 ml) was treated drop wise with *o*-toluidine (0.01 mol) in toluene (20 ml) with constant stirring. The resulting mixture was stirred for one hour and set aside for an additional hour at room temperature for completion of the reaction. The mixture was then treated with dilute hydrochloric acid to remove unreacted *o*-toluidine. The resultant solid *N*-(2-methylphenyl)succinamic acid was filtered under suction and washed thoroughly with water to remove the unreacted succinic anhydride and succinic acid. The compound was recrystallized to constant melting point from ethanol. The purity of the compound was checked by elemental analysis and characterized by its infrared and NMR spectra.

The *N*-(2-methylphenyl)succinamic acid obtained was then treated with phosphorous oxychloride and excess of 3-chloroaniline at room temperature with constant stirring. The resultant mixture was stirred for 4 h, kept aside for additional 6 h for completion of the reaction and poured slowly into crushed ice with constant stirring. It was kept aside for a day. The resultant solid, *N*-(3-chlorophenyl)-*N*'-(2-methylphenyl)succinamide monohydrate was filtered under suction, washed thoroughly with water, with a dilute sodium hydroxide solution and finally with water. It was recrystallized to constant melting point from an acetone/chloroform (1:1 *v*/*v*) solution. The purity of the compound was checked by elemental analysis, and characterized by its infrared and NMR spectra. Needle-like colourless single crystals used for the X-ray diffraction studies were grown by slow evaporation of an acetone/chloroform (1:1 *v*/*v*) solution at room temperature.

### Refinement

The amine and water H atoms were located in a difference Fourier map and refined with the N—H and O—H distances restrained to 0.86 (2) and 0.85 (2) Å, respectively. All other H atoms were positioned with idealized geometry using a riding model with the aromatic C—H = 0.93 Å, methyl C—H = 0.97 mÅ and methylene C—H = 0.97 Å, and with isotropic displacement parameters set to 1.2 times of the *U*_eq_ of the parent atoms. The crystals available for X-ray analysis were of rather poor quality and weak scatterers at high theta value, resulting in relatively high *R* values.

### Crystal data

**Table:**

C_17_H_17_ClN_2_O_2_·H_2_O	*F*(000) = 704
*M**_r_* = 334.79	*D*_x_ = 1.329 Mg m^−^^3^
Monoclinic, *P*2_1_/*c*	Mo *K*α radiation, λ = 0.71073 Å
Hall symbol: -P 2ybc	Cell parameters from 1620 reflections
*a* = 14.875 (4) Å	θ = 2.7–27.9°
*b* = 13.908 (3) Å	µ = 0.24 mm^−^^1^
*c* = 8.088 (2) Å	*T* = 293 K
β = 90.11 (2)°	Needle, colourless
*V* = 1673.3 (7) Å^3^	0.44 × 0.12 × 0.08 mm
*Z* = 4	

### Data collection

**Table:**

Oxford Diffraction Xcalibur diffractometer with a Sapphire CCD detector	3080 independent reflections
Radiation source: fine-focus sealed tube	1436 reflections with *I* > 2σ(*I*)
graphite	*R*_int_ = 0.074
Rotation method data acquisition using ω scans	θ_max_ = 25.5°, θ_min_ = 2.7°
Absorption correction: multi-scan (*CrysAlis RED*; Oxford Diffraction, 2009)	*h* = −18→14
*T*_min_ = 0.900, *T*_max_ = 0.981	*k* = −16→16
6273 measured reflections	*l* = −9→8

### Refinement

**Table:**

Refinement on *F*^2^	Primary atom site location: structure-invariant direct methods
Least-squares matrix: full	Secondary atom site location: difference Fourier map
*R*[*F*^2^ > 2σ(*F*^2^)] = 0.115	Hydrogen site location: inferred from neighbouring sites
*wR*(*F*^2^) = 0.285	H atoms treated by a mixture of independent and constrained refinement
*S* = 1.14	*w* = 1/[σ^2^(*F*_o_^2^) + (0.104*P*)^2^ + 1.4616*P*] where *P* = (*F*_o_^2^ + 2*F*_c_^2^)/3
3080 reflections	(Δ/σ)_max_ = 0.010
221 parameters	Δρ_max_ = 0.66 e Å^−^^3^
5 restraints	Δρ_min_ = −0.27 e Å^−^^3^

### Special details

**Table:**

Experimental. CrysAlis RED (Oxford Diffraction, 2009) Empirical absorption correction using spherical harmonics, implemented in SCALE3 ABSPACK scaling algorithm.
Geometry. All e.s.d.'s (except the e.s.d. in the dihedral angle between two l.s. planes) are estimated using the full covariance matrix. The cell e.s.d.'s are taken into account individually in the estimation of e.s.d.'s in distances, angles and torsion angles; correlations between e.s.d.'s in cell parameters are only used when they are defined by crystal symmetry. An approximate (isotropic) treatment of cell e.s.d.'s is used for estimating e.s.d.'s involving l.s. planes.
Refinement. Refinement of *F*^2^ against ALL reflections. The weighted *R*-factor *wR* and goodness of fit *S* are based on *F*^2^, conventional *R*-factors *R* are based on *F*, with *F* set to zero for negative *F*^2^. The threshold expression of *F*^2^ > σ(*F*^2^) is used only for calculating *R*-factors(gt) *etc*. and is not relevant to the choice of reflections for refinement. *R*-factors based on *F*^2^ are statistically about twice as large as those based on *F*, and *R*- factors based on ALL data will be even larger.

### Fractional atomic coordinates and isotropic or equivalent isotropic displacement parameters (Å^2^)

**Table:**

	*x*	*y*	*z*	*U*_iso_*/*U*_eq_	
Cl1	0.24505 (16)	0.43031 (14)	0.5534 (3)	0.0888 (8)	
O1	0.4988 (3)	0.2577 (3)	0.2806 (6)	0.0588 (13)	
O2	0.6939 (3)	0.1531 (3)	0.3675 (5)	0.0509 (12)	
N1	0.4157 (4)	0.1301 (4)	0.3620 (7)	0.0526 (14)	
H1N	0.414 (4)	0.0680 (15)	0.367 (8)	0.063*	
N2	0.7588 (4)	0.2301 (4)	0.1544 (6)	0.0511 (15)	
H2N	0.757 (4)	0.246 (4)	0.052 (3)	0.061*	
C1	0.3450 (4)	0.1731 (5)	0.4498 (8)	0.0480 (17)	
C2	0.3334 (4)	0.2716 (4)	0.4599 (8)	0.0497 (17)	
H2	0.3746	0.3134	0.4120	0.060*	
C3	0.2595 (5)	0.3062 (5)	0.5426 (9)	0.0571 (19)	
C4	0.1978 (5)	0.2489 (6)	0.6174 (9)	0.0622 (19)	
H4	0.1488	0.2749	0.6729	0.075*	
C5	0.2105 (5)	0.1503 (5)	0.6079 (10)	0.072 (2)	
H5	0.1695	0.1093	0.6582	0.087*	
C6	0.2826 (5)	0.1127 (5)	0.5254 (9)	0.062 (2)	
H6	0.2900	0.0464	0.5198	0.074*	
C7	0.4836 (4)	0.1708 (5)	0.2793 (8)	0.0446 (16)	
C8	0.5394 (4)	0.1008 (5)	0.1842 (8)	0.0542 (18)	
H8A	0.5022	0.0715	0.0994	0.065*	
H8B	0.5591	0.0502	0.2585	0.065*	
C9	0.6221 (5)	0.1455 (5)	0.1022 (8)	0.0540 (18)	
H9A	0.6479	0.0991	0.0263	0.065*	
H9B	0.6033	0.2009	0.0380	0.065*	
C10	0.6931 (4)	0.1764 (4)	0.2227 (8)	0.0404 (15)	
C11	0.8362 (5)	0.2665 (5)	0.2395 (7)	0.0478 (17)	
C12	0.9000 (5)	0.2044 (5)	0.3062 (8)	0.059 (2)	
C13	0.9760 (5)	0.2453 (7)	0.3794 (9)	0.073 (2)	
H13	1.0195	0.2058	0.4264	0.087*	
C14	0.9870 (6)	0.3428 (8)	0.3827 (10)	0.083 (3)	
H14	1.0384	0.3685	0.4312	0.100*	
C15	0.9247 (6)	0.4031 (6)	0.3169 (11)	0.081 (3)	
H15	0.9335	0.4693	0.3196	0.097*	
C16	0.8483 (6)	0.3649 (5)	0.2460 (9)	0.066 (2)	
H16	0.8047	0.4056	0.2024	0.079*	
C17	0.8932 (6)	0.0992 (6)	0.2956 (10)	0.087 (3)	
H17A	0.8438	0.0775	0.3621	0.131*	
H17B	0.9479	0.0708	0.3352	0.131*	
H17C	0.8834	0.0807	0.1827	0.131*	
O3	0.5938 (4)	0.0811 (3)	0.6426 (6)	0.0690 (15)	
H31O	0.614 (5)	0.098 (5)	0.549 (5)	0.104*	
H32O	0.561 (5)	0.125 (4)	0.679 (8)	0.104*	

### Atomic displacement parameters (Å^2^)

**Table:**

	*U*^11^	*U*^22^	*U*^33^	*U*^12^	*U*^13^	*U*^23^
Cl1	0.0963 (17)	0.0478 (12)	0.1224 (19)	0.0147 (12)	0.0074 (14)	−0.0092 (12)
O1	0.062 (3)	0.036 (3)	0.079 (3)	−0.011 (2)	0.007 (2)	−0.005 (2)
O2	0.054 (3)	0.055 (3)	0.044 (3)	0.002 (2)	0.006 (2)	0.005 (2)
N1	0.047 (3)	0.036 (3)	0.074 (4)	−0.002 (3)	0.006 (3)	0.004 (3)
N2	0.059 (4)	0.056 (4)	0.039 (3)	0.004 (3)	0.006 (3)	0.005 (3)
C1	0.046 (4)	0.046 (4)	0.052 (4)	−0.007 (3)	−0.006 (3)	0.001 (3)
C2	0.044 (4)	0.042 (4)	0.062 (4)	−0.001 (3)	−0.001 (4)	−0.001 (3)
C3	0.059 (5)	0.048 (4)	0.065 (4)	−0.001 (4)	−0.006 (4)	−0.008 (4)
C4	0.053 (4)	0.069 (5)	0.066 (5)	0.005 (4)	0.007 (4)	−0.003 (4)
C5	0.067 (5)	0.059 (5)	0.091 (6)	−0.009 (4)	0.017 (5)	0.003 (4)
C6	0.064 (5)	0.048 (4)	0.075 (5)	−0.004 (4)	0.013 (4)	−0.001 (4)
C7	0.040 (4)	0.042 (4)	0.052 (4)	−0.002 (3)	−0.002 (3)	−0.002 (3)
C8	0.055 (4)	0.040 (4)	0.067 (4)	−0.007 (3)	−0.006 (4)	−0.011 (3)
C9	0.068 (5)	0.048 (4)	0.046 (4)	0.004 (4)	0.003 (4)	−0.010 (3)
C10	0.049 (4)	0.036 (3)	0.036 (4)	0.004 (3)	0.004 (3)	−0.007 (3)
C11	0.047 (4)	0.058 (4)	0.039 (4)	0.002 (4)	0.002 (3)	−0.002 (3)
C12	0.062 (5)	0.058 (5)	0.056 (4)	0.002 (4)	0.019 (4)	0.002 (4)
C13	0.046 (5)	0.098 (7)	0.073 (5)	0.002 (5)	0.000 (4)	−0.008 (5)
C14	0.057 (5)	0.111 (8)	0.082 (6)	−0.019 (6)	0.007 (5)	−0.016 (6)
C15	0.082 (6)	0.061 (5)	0.099 (7)	−0.014 (5)	0.008 (6)	−0.006 (5)
C16	0.069 (5)	0.051 (5)	0.076 (5)	−0.009 (4)	0.007 (4)	−0.006 (4)
C17	0.089 (7)	0.071 (6)	0.102 (7)	0.010 (5)	0.014 (5)	0.000 (5)
O3	0.091 (4)	0.034 (3)	0.083 (4)	0.008 (3)	0.028 (3)	0.008 (3)

### Geometric parameters (Å, °)

**Table:**

Cl1—C3	1.742 (7)	C8—H8A	0.9700
O1—C7	1.230 (7)	C8—H8B	0.9700
O2—C10	1.215 (7)	C9—C10	1.499 (9)
N1—C7	1.338 (8)	C9—H9A	0.9700
N1—C1	1.404 (8)	C9—H9B	0.9700
N1—H1N	0.86 (2)	C11—C16	1.381 (9)
N2—C10	1.350 (8)	C11—C12	1.391 (9)
N2—C11	1.432 (8)	C12—C13	1.396 (10)
N2—H2N	0.86 (2)	C12—C17	1.469 (10)
C1—C2	1.384 (9)	C13—C14	1.366 (11)
C1—C6	1.393 (9)	C13—H13	0.9300
C2—C3	1.375 (9)	C14—C15	1.357 (11)
C2—H2	0.9300	C14—H14	0.9300
C3—C4	1.358 (9)	C15—C16	1.378 (11)
C4—C5	1.386 (9)	C15—H15	0.9300
C4—H4	0.9300	C16—H16	0.9300
C5—C6	1.368 (10)	C17—H17A	0.9600
C5—H5	0.9300	C17—H17B	0.9600
C6—H6	0.9300	C17—H17C	0.9600
C7—C8	1.494 (9)	O3—H31O	0.85 (2)
C8—C9	1.531 (9)	O3—H32O	0.84 (2)
			
C7—N1—C1	129.7 (6)	C10—C9—H9A	108.9
C7—N1—H1N	118 (4)	C8—C9—H9A	108.8
C1—N1—H1N	113 (4)	C10—C9—H9B	108.8
C10—N2—C11	125.5 (5)	C8—C9—H9B	108.8
C10—N2—H2N	121 (5)	H9A—C9—H9B	107.7
C11—N2—H2N	113 (5)	O2—C10—N2	122.4 (6)
C2—C1—C6	119.2 (6)	O2—C10—C9	123.8 (6)
C2—C1—N1	123.0 (6)	N2—C10—C9	113.7 (5)
C6—C1—N1	117.8 (6)	C16—C11—C12	120.8 (7)
C3—C2—C1	118.3 (6)	C16—C11—N2	118.2 (6)
C3—C2—H2	120.8	C12—C11—N2	120.9 (6)
C1—C2—H2	120.8	C11—C12—C13	117.6 (7)
C4—C3—C2	123.6 (7)	C11—C12—C17	123.3 (7)
C4—C3—Cl1	118.4 (6)	C13—C12—C17	119.1 (8)
C2—C3—Cl1	118.0 (6)	C14—C13—C12	120.6 (8)
C3—C4—C5	117.6 (7)	C14—C13—H13	119.7
C3—C4—H4	121.2	C12—C13—H13	119.7
C5—C4—H4	121.2	C15—C14—C13	121.6 (9)
C6—C5—C4	120.8 (7)	C15—C14—H14	119.2
C6—C5—H5	119.6	C13—C14—H14	119.2
C4—C5—H5	119.6	C14—C15—C16	119.1 (8)
C5—C6—C1	120.5 (7)	C14—C15—H15	120.4
C5—C6—H6	119.7	C16—C15—H15	120.4
C1—C6—H6	119.7	C15—C16—C11	120.3 (8)
O1—C7—N1	123.5 (6)	C15—C16—H16	119.8
O1—C7—C8	122.8 (6)	C11—C16—H16	119.8
N1—C7—C8	113.7 (6)	C12—C17—H17A	109.5
C7—C8—C9	114.0 (5)	C12—C17—H17B	109.5
C7—C8—H8A	108.8	H17A—C17—H17B	109.5
C9—C8—H8A	108.8	C12—C17—H17C	109.5
C7—C8—H8B	108.8	H17A—C17—H17C	109.5
C9—C8—H8B	108.8	H17B—C17—H17C	109.5
H8A—C8—H8B	107.7	H31O—O3—H32O	108 (3)
C10—C9—C8	113.6 (5)		
			
C7—N1—C1—C2	2.1 (11)	C11—N2—C10—O2	−0.2 (10)
C7—N1—C1—C6	−180.0 (7)	C11—N2—C10—C9	−177.8 (6)
C6—C1—C2—C3	−1.0 (10)	C8—C9—C10—O2	13.0 (9)
N1—C1—C2—C3	177.0 (6)	C8—C9—C10—N2	−169.4 (5)
C1—C2—C3—C4	1.1 (10)	C10—N2—C11—C16	−119.5 (7)
C1—C2—C3—Cl1	−179.5 (5)	C10—N2—C11—C12	63.9 (8)
C2—C3—C4—C5	−0.4 (11)	C16—C11—C12—C13	0.1 (9)
Cl1—C3—C4—C5	−179.9 (6)	N2—C11—C12—C13	176.6 (6)
C3—C4—C5—C6	−0.2 (12)	C16—C11—C12—C17	−176.8 (7)
C4—C5—C6—C1	0.3 (12)	N2—C11—C12—C17	−0.2 (9)
C2—C1—C6—C5	0.3 (11)	C11—C12—C13—C14	−0.8 (10)
N1—C1—C6—C5	−177.7 (7)	C17—C12—C13—C14	176.2 (7)
C1—N1—C7—O1	6.9 (11)	C12—C13—C14—C15	0.6 (13)
C1—N1—C7—C8	−173.0 (6)	C13—C14—C15—C16	0.4 (13)
O1—C7—C8—C9	5.4 (9)	C14—C15—C16—C11	−1.2 (12)
N1—C7—C8—C9	−174.7 (6)	C12—C11—C16—C15	0.9 (10)
C7—C8—C9—C10	69.5 (7)	N2—C11—C16—C15	−175.7 (6)

### Hydrogen-bond geometry (Å, °)

**Table:**

*D*—H···*A*	*D*—H	H···*A*	*D*···*A*	*D*—H···*A*
N1—H1N···O3^i^	0.86 (2)	2.08 (2)	2.940 (7)	175 (6)
N2—H2N···O2^ii^	0.86 (2)	2.25 (4)	2.991 (7)	145 (6)
O3—H31O···O2	0.85 (2)	2.04 (3)	2.861 (6)	163 (8)
O3—H32O···O1^iii^	0.84 (2)	2.05 (3)	2.877 (6)	171 (9)

Symmetry codes: (i) −*x*+1, −*y*, −*z*+1; (ii) *x*, −*y*+1/2, *z*−1/2; (iii) *x*, −*y*+1/2, *z*+1/2.
